# Neonicotinoid Binding, Toxicity and Expression of Nicotinic Acetylcholine Receptor Subunits in the Aphid *Acyrthosiphon pisum*

**DOI:** 10.1371/journal.pone.0096669

**Published:** 2014-05-06

**Authors:** Emiliane Taillebois, Abdelhamid Beloula, Sophie Quinchard, Stéphanie Jaubert-Possamai, Antoine Daguin, Denis Servent, Denis Tagu, Steeve H. Thany, Hélène Tricoire-Leignel

**Affiliations:** 1 Laboratoire Récepteurs et Canaux Ioniques Membranaires (RCIM), UPRES EA 2647 USC INRA 1330, SFR QUASAV 4207, Université d’Angers, Angers, France; 2 Institut de Génétique Environnement et Protection des Plantes (IGEPP), Institut National de la Recherche Agronomique (INRA), UMR 1349, Le Rheu, France; 3 Groupement Interprofessionnel de Recherche sur les Produits Agropharmaceutiques (GIRPA), Angers, France; 4 Institut de Biologie et Technologie (iBiTecS), Service d’Ingénierie Moléculaire des Protéines (SIMOPRO), Commisariat à l’Energie Atomique (CEA), Gif-sur-Yvette, France; Ghent University, Belgium

## Abstract

Neonicotinoid insecticides act on nicotinic acetylcholine receptor and are particularly effective against sucking pests. They are widely used in crops protection to fight against aphids, which cause severe damage. In the present study we evaluated the susceptibility of the pea aphid *Acyrthosiphon pisum* to the commonly used neonicotinoid insecticides imidacloprid (IMI), thiamethoxam (TMX) and clothianidin (CLT). Binding studies on aphid membrane preparations revealed the existence of high and low-affinity binding sites for [^3^H]-IMI (Kd of 0.16±0.04 nM and 41.7±5.9 nM) and for the nicotinic antagonist [^125^I]-α-bungarotoxin (Kd of 0.008±0.002 nM and 1.135±0.213 nM). Competitive binding experiments demonstrated that TMX displayed a higher affinity than IMI for [^125^I]-α-bungarotoxin binding sites while CLT affinity was similar for both [^125^I]-α-bungarotoxin and [^3^H]-IMI binding sites. Interestingly, toxicological studies revealed that at 48 h, IMI (LC_50_ = 0.038 µg/ml) and TMX (LC_50_ = 0.034 µg/ml) were more toxic than CLT (LC_50_ = 0.118 µg/ml). The effect of TMX could be associated to its metabolite CLT as demonstrated by HPLC/MS analysis. In addition, we found that aphid larvae treated either with IMI, TMX or CLT showed a strong variation of nAChR subunit expression. Using semi-quantitative PCR experiments, we detected for all insecticides an increase of Apisumα10 and Apisumβ1 expressions levels, whereas Apisumβ2 expression decreased. Moreover, some other receptor subunits seemed to be differently regulated according to the insecticide used. Finally, we also demonstrated that nAChR subunit expression differed during pea aphid development. Altogether these results highlight species specificity that should be taken into account in pest management strategies.

## Introduction

Neonicotinoid insecticides include several compounds such as imidacloprid (IMI), clothanidin (CLT) and thiametoxam (TMX). They are efficient agonists of insect neuronal nicotinic acetylcholine receptors (nAChRs) which are pentameric receptors formed by identical (homomeric) or different (heteromeric) subunits [1], [2], [3]. In the context of a stronger legislation on insecticide use to limit environmental and health concerns, approaches to describe and understand the cellular and molecular mechanisms involved in insecticide resistance are needed. Insects represent a very diverse group of animals and most Orders diverged approximately 300 million years ago [4]. As a consequence, adaptive mechanisms that confer insecticide resistance can vary from one order/species to another. Among insect pests that cause damage to agriculture, aphids (Hemiptera) have a particular biology: they feed from phloem sap (not by chewing plant tissues), and thus transmit plant viruses very efficiently [5]. Their pest status is also attributable to their peculiar reproductive mode [5]. Asexual reproduction of aphids by parthenogenesis (during spring and summer) leads to extremely rapid population growth [6]. Several insecticides, such as neonicotinoids, are used as seed treatment to limit the impact of sucking-pest like aphids, *Sitobion avenae, Aphis craccivora* and *Myzus persicae* [7], [8].

Bioassay studies have revealed that neonicotinicotinoid susceptibility varies between insect species [8], [9], [10]. In Hemiptera, IMI showed susceptibility differences with LC_95_ values between 0.32 and 40 mg.L^−1^ [9]. Moreover, studies performed with the cotton aphid *Aphis gossipii* demonstrated that one IMI resistant-strain was still susceptible toward TMX and CLT suggesting no cross resistance [10] whereas a cross resistance was found in *Myzus persicae,* with resistance factors of 11, 18 and 100 for IMI, TMX and CLT, respectively [8]. This discrepancy suggested that some aphid species could carry particular resistance mechanisms. Similar differences could be found using competitive binding studies. In *Aphis craccivora* it was demonstrated that TMX was a non-competitive inhibitor for [^3^H]-IMI suggesting that it binds to a different site or in a different mode than IMI while in *Myzus persicae,* it was found that the resistant strain with the R81T mutation on the Mpβ1 subunit developed cross-resistance against IMI and TMX demonstrating that they interact with the same site [3]. In addition, saturation studies suggest that IMI binds to high- and low-affinity binding sites in the aphids *M. persicae* and *A. craccivora* [11], [12]. Similar high and low affinity binding sites were also identified in *M. persicae* for the nicotinic antagonist, α-Bungarotoxin (α-Bgt) [13]. Altogether these data demonstrate that aphid species can carry different sensitivities against neonicotinoid insecticides.

In this study, we evaluated the binding properties and toxicological effects of IMI, TMX and CLT on the pea aphid and showed that IMI and TMX bind two different nAChR populations, both binding CLT, although the toxicity of CLT is lower than toxicities of IMI and TMX. In addition, using the full sequenced genome of *A. pisum* [14], we demonstrated that the eleven *A. pisum* nAChR subunits previously identified [15] are differentially regulated during aphid development and after neonicotinoid intoxication.

## Materials and Methods

### Insects

The pea aphid (*A.pisum*) sequenced strain LSR1 (corresponding to the reference genome) was generously provided by INRA-Rennes IGEPP. Unwinged parthenogenetic females were reared on faba bean (*Vicia fabae*) plants in a 16L: 8D photoperiod at constant temperature of 22°C in a climate chamber. Under these conditions, aphids reproduce by viviparous parthenogenesis, as clonal female: new born larvae become adults after four molts. Adults and larvae at each of the 4 stages were collected. Larval stages were determined by identifying the number of antennal segments.

### Insecticides

IMI, TMX and CLT were purchased from Sigma-Aldrich (Saint-Louis, USA). Insecticides were dissolved in DMSO to give final concentrations of 50 mg.ml^−1^. For binding experiments, insecticides were dissolved in the corresponding buffer (PBS buffer or Tris-HCl buffer for [^125^I]-α-Bgt and [^3^H]-IMI experiments respectively). For intoxication experiments, insecticides were dissolved in artificial diet at a final concentration of 0.2% DMSO for 100µg/ml and 2% DMSO for 1000 µg/ml. These concentrations of DMSO were used as controls in insecticidal assays.

### Binding assays

Membrane preparations were isolated from frozen aphids according to the Wiesner and Kayser protocol [12]. Whole aphids were homogenized with a pestle motor in 4°C dissociation medium at pH 7.0. The dissociation medium contained: 20 mM sodium phosphate, 150 mM sodium chloride, 1 mM EDTA, 0.1 mM phenylmethanesulfonyl fluoride (dissolved in acetone), and 2 µg each of pepstatin, chymostatin, and leupeptin (dissolved in methanol, DMSO, and water, respectively). After homogenization, samples were centrifuged 10 min at 1000 g and supernatant was collected and ultracentrifuged 30 min at 4°C and 43000 g. The precipitate was washed with cold dissociation medium and then ultracentrifuged. The final pellet was resuspended in 3 ml of dissociation medium. Total protein was quantified by spectrofluorometry at 750 nm according to the Lowry colorimetric method (DC protein assay, Biorad, France) with a range of BSA as a standard. Membrane preparations were conserved at −80°C until use.

α-Bgt binding experiments were performed using 40 µg of aphid total membrane protein in a total volume of 300 µl of PBS Buffer (Na2HPO4, NaH2PO4, NaCl, pH = 7.2) + 0.1% of bovine serum albumin and [^125^I]-α-Bgt (2200 Ci/mmol, PerkinElmer, USA) as radiolabeled ligand for total binding measurement. For non-specific binding determination, 1 µM of cobratoxin was added prior to membrane incubation. In saturation assays, the concentration of [^125^I]-α-Bgt varied from 18 nM to 0.9 pM to obtain a complete saturation binding curve. Competitive assays were performed with IMI, TMX and CLT. For this purpose, membranes were incubated with various concentrations of unlabeled competitor and [^125^I]-α-Bgt at 0.08 nM for CLT and TMX and 0.6 nM for IMI, respectively. Incubations were performed at room temperature during 4 hours and terminated by rapid vacuum filtration using GF/C Glass microfiber filters presoaked in polyethyleneiminine 0.5%. Filters were rapidly washed (< 20 s) twice with 5 ml of cold PBS Buffer at 0.01 M and transferred in tubes for immediate counting on a γ-counter.

IMI binding experiments were performed using 200 µg of aphid total membrane protein in a final volume of 300 µl of Tris-HCl Buffer (10 mM, pH = 7.4) and [^3^H]-IMI (ARC, 40 Ci/mmol) as radiotracer for total binding measurement. For non-specific binding determination, 0.3 mM of unlabeled IMI was added prior to membrane incubation. In saturation assays, the concentration of [^3^H]-IMI varied from 500 nM to 0.5 pM to obtain a complete binding curve. Competitive assays were achieved for CLT, TMX, IMI and α-Bgt using 25 nM of [^3^H]-IMI. Incubations were performed at room temperature during 4 hours and terminated by rapid vacuum filtration using GF/C Glass microfiber filters presoaked in 0.5% polyethyleneiminine. Filters were rapidly washed (< 20s) with cold Tris-HCl Buffer and dried for 1 hour before incubation in 5 ml of scintillation liquid (PerkinElmer, USA) and counting.

### Insecticidal assays

The susceptibility of *A. pisum* to IMI, TMX and CLT was determined using an artificial diet bioassay according to Sadeghi *et al*.[16]. In brief, adults were put on a feeding apparatus (day D-1) containing 200 µl of artificial diet. Then, the first-instar nymphs were transferred (day D0) to freshly prepared diet with insecticide added (treatment series) or DMSO added (control series). For each insecticide, eight concentrations ranging from 0.001 to 1000 µg.ml^−1^ were tested. The mortality was scored after 24 h (day D+1) and 48 h (day D+2). Aphids that were unable to walk were considered dead [8] and were removed. Corrected mortality percentages were calculated using Henderson Tilton's formula after 24 h (day D+1) and 48 h (day D+2) of insecticide exposure.

### HPLC-MS/MS analysis

For tissue extraction, 2.4 g of TMX (at LC_50_) treated aphid larvae were extracted with an acidified (0.2% acetic acid) water-methanol mixture (50/50) in a 50 ml centrifuge tube. Sample extracts were then filtered and purified on Oasis HLB Cartridges (200 mg) (Waters SAS, France). Elution was performed using 6 ml of acetonitrile. The obtained acetonitrile extract after the elution of the Oasis cartridges was reduced to dryness and the residue was re-dissolved in 1 ml methanol-water mixture (10/90). 40 µl of each sample was analyzed by high-performance liquid chromatography coupled to tandem mass spectrometry (HPLC-MS/MS).

HPLC–MS/MS was performed with an Ultimate 3000 rapid separation liquid chromatography system (Dionex, USA) coupled to an API 4000 Qtrap MS/MS from Applied Biosystems (Foster City, CA, USA). Separation was performed on a Phenomenex (Torrance, CA, USA) C18 column at 35°C with a gradient of water/methanol/acetic acid at a flow rate of 0.2 ml.min^−1^ and 5 mM ammonium acetate. MS/MS detection was performed in the multi-reaction-monitoring (MRM) mode using an ESI interface in the positive ion mode. The ionization voltage was 5500V, and the nebulizer and curtain gases were at 50 psi and 25 psi, respectively. The drying gas to assist the solvent evaporation in the source (600°C) was at 40 psi.

Optimisation of MRM transitions, collision energies and cone voltage were performed by direct injection of standard solutions. The optimized parameters for the detection of the two compounds (TMX and CLT) are listed in Table 1. With these parameters, calibration curves were linear over the concentration range of 0.9 to 20 µg.l^−1^ with a correlation coefficient (r) greater than 0.99.

**Table 1:** Multireaction monitoring conditions used for the HPLC-MS/MS analysis.

Compound	Ion	Transition	Declustering potential (DP)	Collision energy (CE)	Collision cell exit potential (CXP)	Dwell time (s)	HPLC retention time (tr)(min)	
clothianidin	[M+H]^+^	250>169	46	19	8	250		4,19
clothianidin	[M+H]^+^	250>132	46	21	10	250		
thiamethoxam	[M+H]^+^	292>211	56	17	10	250		3,84
thiamethoxam	[M+H]^+^	292>181	56	33	8	250		

### Expression of nAChR subunits during developmental stages

Total RNAs were extracted from *A. pisum* adults, at different larval stages (Stage L1 to L4), using RNA Easy mini Plant Kit (Qiagen, Courtaboeuf France). To avoid genomic DNA (gDNA) contamination, total RNAs were treated using DNAse I kit (Invitrogen, Carlsbad, USA) according to manufacturer recommendations. RNAs were retro-transcribed using random hexamers with RevertAid kit (Thermoscientific, Waltham, USA), dissolved in RNAse-free water and conserved at −20°C. DNAse treatment was validated by PCR using primers set amplifying intron-containing sequence. Primer sets (Table 2) were designed using Primer3 software based on the *A. pisum* genome (http://www.ncbi.nlm.nih.gov/genbank/). Because Dale et al. identified potential alternatively spliced isoforms for Apisumα4 (exon 4) Apisumα6 (exon 6) and Apisumα7 (exon 6 and 7), primers were designed out of these exons, using genome information [14]. Amplification specificity of each primer set was also verified by cloning and sequencing the amplification products (data not shown). Amplification efficiencies were between 88 and 109%, allowing validation of each primer set for qPCR experiments. Because none of the endogenous reference genes had stable expression during developmental stages, external reference gene, *luciferase,* was used for normalization, as previously described [17], [18]. Thus, 10 pg/1000 ng of luciferase RNA (Promega, Fitchburg, Wisconsin USA) were added after RNA extraction [17], [18].

**Table 2:** Primers used to amplify nicotinic acetylcholine receptors subunits in quantitative PCR experiments.

gene	forward primer		reverse primer		Size (bp)
	name	nucleotidic sequence	name	nucleotidic sequence	
α 1	qpA1S1	CGGTCATTGTCGGTCAGTTG	qpA1R1	TGGCATCGGCACTTCCAT	60
α 2	qpA2S2	GGTCGTCACCATCATCATC	qpA2R2	CCACGACGGTATCTTGTGC	68
α 3	qpA3S1	GCGAGATTCACGGTCCAATAA	qpA3R1	GGCCATTTTGGTTTGTTTCG	60
α 4	qpA4S1	GAGTATGGTGGCGTGCAAATG	qpA4R1	GATATCCGGCCGCCAAAT	60
α 6	qpA6S1	TGGAGAGACCTGTATCCAACGA	qpA6R1	TGCTGTAGCGTGATGCCAAA	64
α 7	qpA7S1	CATGTATAATAGCGCTGACGAAGGT	qpA7R1	CTGTTGACCACCACGTTGGTT	63
α 8	qpA8S1	GAGGCACATCGACCAATCG	qpA8R1	CGCTTAGATCAATGCCAACATC	59
α 9	qpA9S1	GTGCAACCCGTGCAGTACAG	qpA9R1	TGCGTGTCATACGGCCAATA	65
α 10	qpA10S1	GCACATGGTTCATAGCGAACTG	qpA10R1	GGTGTTCATATTCGCTCGGATT	66
β1	qpB11S1	CGCCGTCCAAACACAAGAT	qpB11R1	CTTGCAGTTGGGATGATGCA	62
β2	qpB2S1	CCGTGAAGAGGAAAATACCG	qpB2R1	GAACACGACGACTATCGCTG	65
rpl7	qpRPL7F	GCGCGCCGAGGCTTAT	qpRPL7R	CCGGATTTCTTTGCATTTCTTG	81
actin	qpactinF	AGCTCTATTCCAACCTTCCTTCT	qpactinR	TGTATGTAGTCTCGTGGATACCG	62

qPCR experiments were optimized according to MIQE Guideline recommendations [19] using ABI Prism 7700 instrument and 2X SYBR Green PCR Master Mix (Applied Biosystems, Courtaboeuf, France). Experiments were performed in triplicate using 100 ng of total RNA and 150 nM primers in a final volume of 25 µl. Product specificity was further assessed by electrophoresis on a 2% agarose gel with a 50 bp ladder and by dissociation curves giving rise to a single peak at the specific melting temperature [20]. Relative expression ratio (R) was calculated according to the Pfaffl formula [21], using primer efficiency (E) and CP value variation between control and sample (ΔCP) for each nAChR subunit. Ratio were normalized to reference genes and expressed in percentage: R  =  (E_subunit_)^ΔCPsubunit(control – sample)^ / (E_reference_)^ΔCPreference (control – sample)^. Luciferase was used as reference gene and quantification was relative to the first larval stage (L1).

### Expression of nAChR subunits after neonicotinoid intoxication

To study subunit expression levels after insecticide exposure, relative qPCR was performed on first larval stage L1 exposed during 48 h with each neonicotinoid at LC_50_ or with DMSO (control condition). Total RNAs were extracted from intoxicated or control first larval stage (L1) using RNA Easy mini plant Kit and the same primers as described above. The results were normalized using the geometric mean of two reference genes, *actin* and *rpl7* and validated using Normfinder software [22], [23], [24]. Expression levels were relative to control condition.

### Statistical analyses

Statistical analyses were performed using GraphPad Prism 5 (GraphPad Software Inc., La Jolla, CA). Data from binding experiments and insecticidal assays were analyzed by nonlinear regression analysis. A T-test (P<0.05; t-test with Welch's correction) was used for insecticidal assays and to compare Ki values. One-Way ANOVA (p<0.05) was used for qPCR experiments and binding assays.

## Results

### Binding properties of IMI, TMX and CLT on *A.pisum* native nAChRs

**Figure 1:**
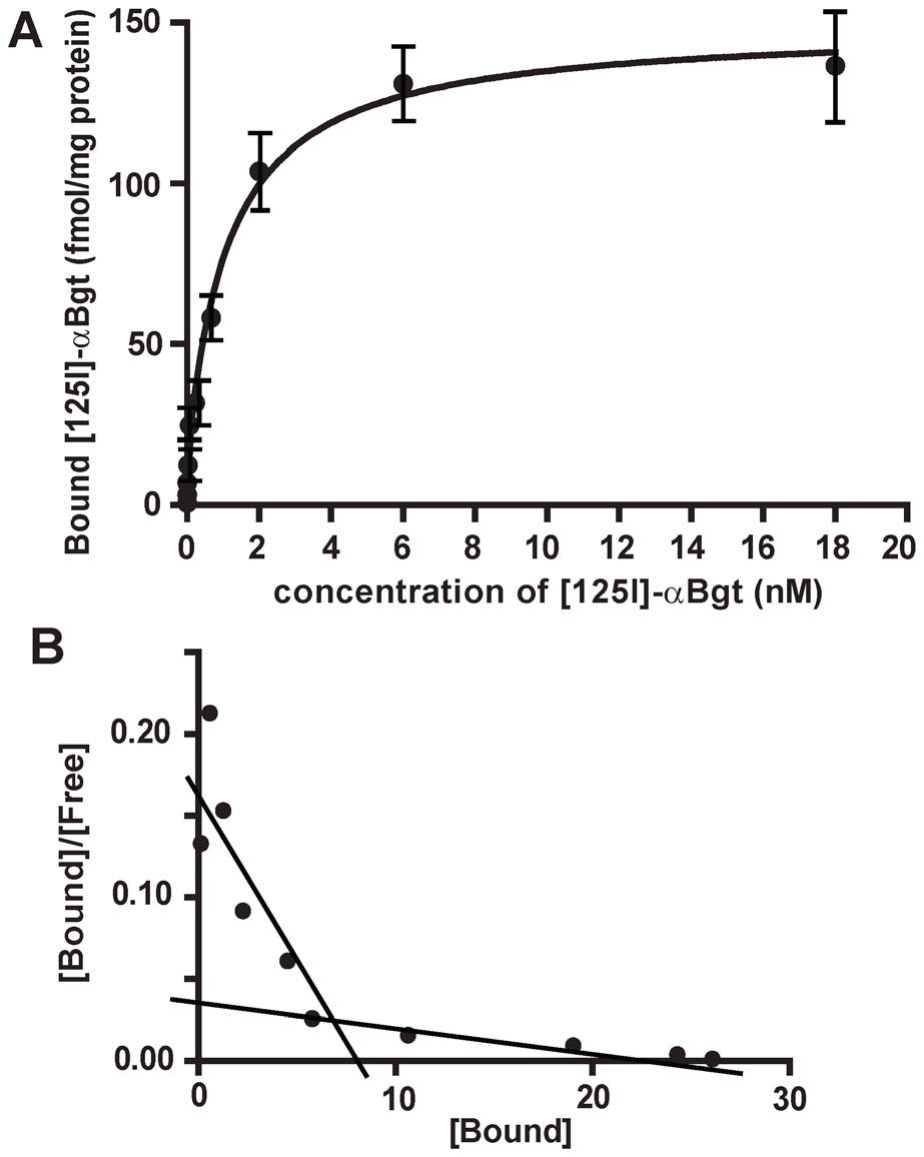
[^125^I]-α-Bungarotoxin specific binding on pea aphid. Saturation curves (A) and Scatchard plots (B) for [^125^I]-α-Bungarotoxin (α-Bgt) specific binding. Membranes were extracted from whole parthenogenetic adults of pea aphid *Acyrthosiphon pisum* LSR1. Results are means of four experiments. Error bars represent the SEM.

**Figure 2:**
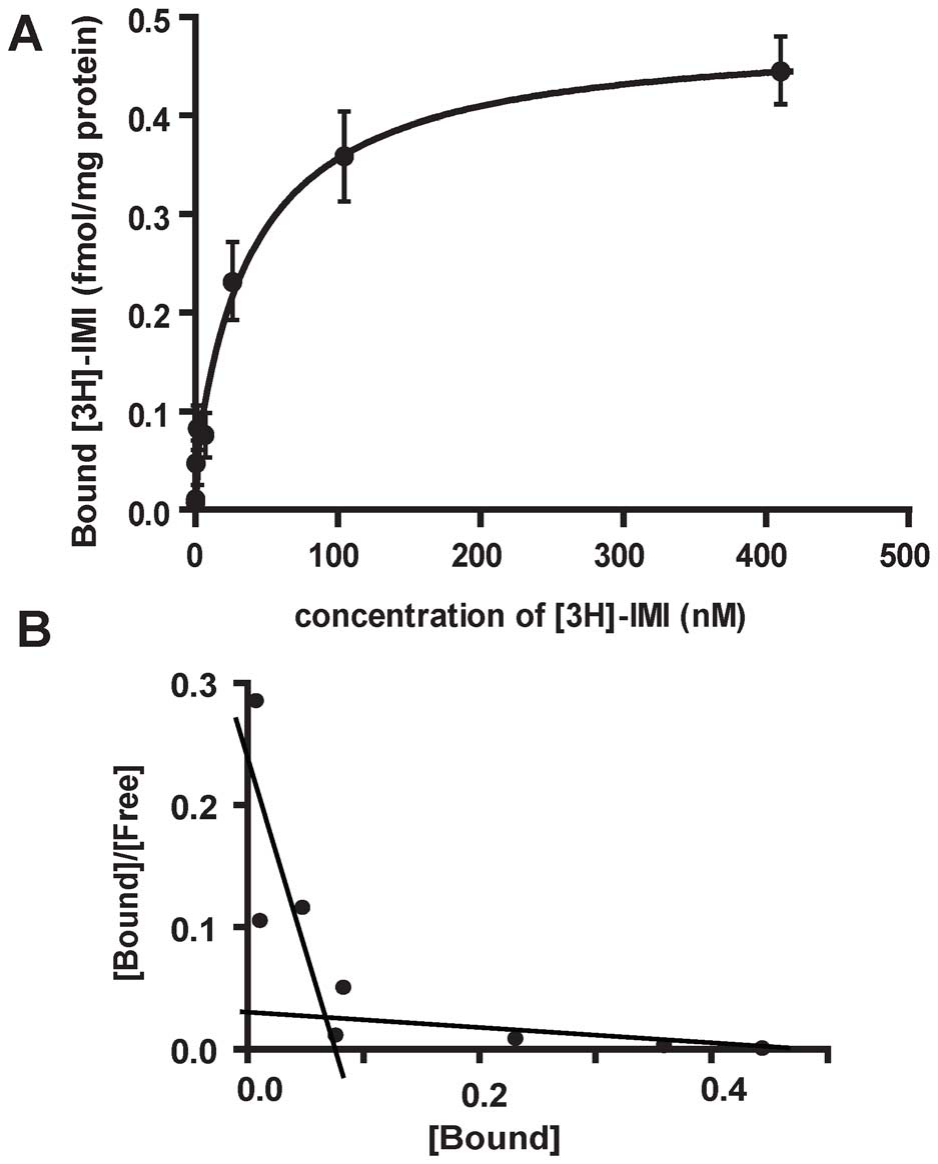
[^3^H]-imidacloprid specific binding on pea aphid. Saturation curves (A) and Scatchard plots (B) for [^3^H]-imidacloprid specific binding. Membranes were extracted from whole parthenogenetic adults of pea aphid *Acyrthosiphon pisum* LSR1. Results are means of four experiments. Error bars represent the SEM.

Saturation binding experiments were carried out with both [^125^I]-α-Bgt and [^3^H]-IMI on adult aphid membranes. Results are means of four experiments and the saturation binding parameters are summarized in Table 3. A saturation curve was first determined for [^125^I]-α-Bgt (Figure 1A and 1B) and revealed the presence of two binding sites: a high affinity (Kd  =  0.008±0.002 nM and Bmax  =  12.86±5.92 fmol/mg protein) and a low-affinity binding site (Kd  =  1.135±0.213 nM and Bmax  =  135.9±6.0 fmol/mg protein). The saturation data obtained for [^3^H]-IMI (Figure 2A and 2B) were also consistent with the presence of a high affinity (Kd  =  0.16±0.04 nM and Bmax  =  0.051±0.003 fmol/mg protein) and a low-affinity binding site (Kd  =  41.7±5.9 nM and Bmax  =  0.434±0.037 fmol/mg protein). For both [^3^H]-IMI and [^125^I]-α-Bgt saturations curves, the presence of two binding sites was supported by the slope change in the Scatchard representation (Figure 1B and 2B). In addition, we noticed that high affinity binding sites only represented 8.6±3.8% and 10.4±5.2% of total [^125^I]-α-Bgt and [^3^H]-IMI binding sites, respectively (Table 3). The difference between Bmax values for high- and low-affinity binding sites, for both [^125^I]-α-Bgt and [^3^H]-IMI, is consistent with the presence of these two binding sites on different nAChR populations in the aphid membrane preparation. In addition, the comparison of Bmax values highlighted a larger proportion of [^125^I]-α-Bgt binding sites compared to [^3^H]-IMI (Table 3). Thus in the pea aphid *A. pisum*, α-Bgt-sensitive nAChRs seem to represent a large majority of nAChR populations.

**Table 3:** [^125^I]-α-Bungarotoxin ([^125^I]-α-Bgt) and [^3^H]-imidacloprid ([^3^H]-IMI) binding parameters determined on aphid membranes.

Saturation binding						
	[^125^I]-α-Bgt			[^3^H]-IMI		
	Kd (nM)	Bmax (fmol/mg)	% of high affinity	Kd (nM)	Bmax (fmol/mg)	% of high affinity
High affinity	0.008±0.002	12.86±5.92	8.6±3.8	0.16±0.04	0.051±0.003	10.4±5.2
Low affinity	1.135±0.213	135.9±6.0		41.7±5.9	0.434±0.037	

Competition binding						
	[^125^I]- α-Bgt			[^3^H]-IMI		
	assay level (nM)	IC50 (μM)	Ki (nM)	assay level (nM)	IC50 (μM)	Ki (nM)
Imidacloprid	0.6	22.33±1.73	14.6±1.13^a^	25	0.061±0.011	38.14±6.88^c^
Thiamethoxam	0.08	1.636±0.701	1.53±0.65^a^	25	1.675±0.101	1047.2±63.1^d^
Clothianidin	0.08	0.194±0.052	0.18±0.05^b^	25	0.203±0.068	126.9±42.5^c^
α-Bgt	-	-	-	25	n.d.	n.d.

Ki values were calculated according to Cheng and Prusoff formula considering Kd of low affinity binding sites. n.d: not determined. IC50: half maximal inhibitory concentration. Results are mean of four experiments and are represented ± SD. Ki values that are significantly different using One-Way ANOVA (p<0.05) are noted with different letters.

**Figure 3:**
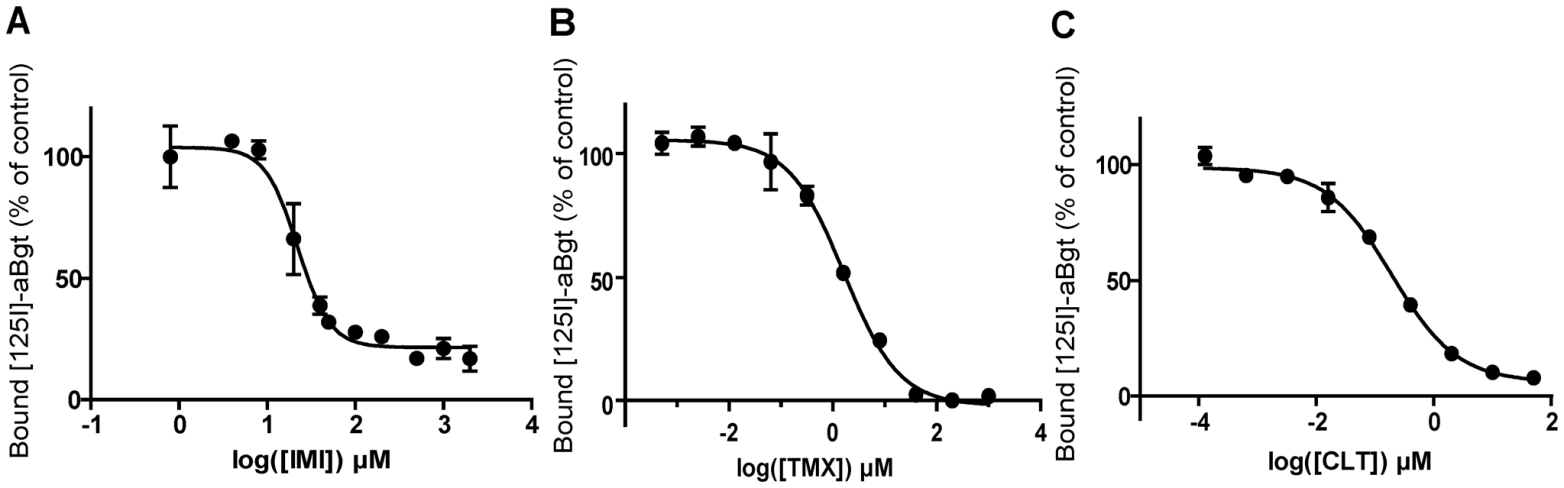
Neonicotinoids inhibition of [^125^I]-α-Bungarotoxin specific binding. Inhibition curves were determined on membranes of whole parthenogenetic adults of pea aphid *Acyrthosiphon pisum* for three neonicotinoids: A) imidacloprid (IMI), B) thiamethoxam (TMX) and C) clothianidin (CLT). Results are means of four experiments. Error bars represent the SEM.

**Figure 4:**
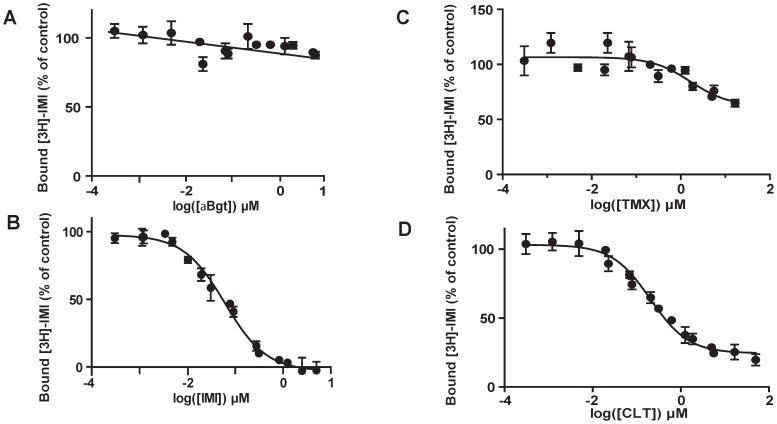
Neonicotinoid inhibition of [^3^H]-imidacloprid specific binding. Inhibition curves were determined on membranes of whole parthenogenetic adults of pea aphid *Acyrthosiphon pisum* for (A) α-Bungarotoxin (α-Bgt) and three neonicotinoids: (B) imidacloprid (IMI), (C) thiamethoxam (TMX) and (D) clothianidin (CLT). Results are means of four experiments. Error bars represent the SEM.

In a second set of experiments, we studied the binding properties of IMI, TMX and CLT to the different nAChRs. Results are means of four experiments and the competition binding parameters are summarized in Table 3. For both radiotracers ([^3^H]-IMI and [^125^I]-α-Bgt), we studied the low affinity binding sites, which represent the majority of nAChR subtypes. Inhibition curves with IMI showed the presence of 20% of [^125^I]-α-Bgt residual binding in excess of IMI, suggesting that some of the α-Bgt binding sites were insensitive to IMI (Figure 3A). On the contrary, inhibition was complete using TMX (Figure 3B) and CLT (Figure 3C). Indeed, the inhibition constant (Ki) for α-Bgt with a Kd value of 0.16 nM showed a better binding affinity for CLT and TMX (Ki  =  0.18±0.05 µM and 1.53±0.65 µM, respectively) compared to IMI (Ki  =  14.61±1.13 µM; One-Way ANOVA, p<0.05, table 3). With [^3^H]-IMI, no specific binding inhibition was found using α-Bgt, indicating that low-affinity [^3^H]-IMI binding sites were insensitive to α-Bgt (Figure 4A). Among the tested insecticides, homologous competition (Figure 4B) showed that IMI presents a Ki value of 38.14±6.88 nM which is consistent with the Kd value determined in the saturation experiment (41.7 nM, table 3). Interestingly, high concentrations of TMX and CLT were not able to completely displace [^3^H]-IMI from its binding sites, with maximal inhibition of 35% for TMX (Figure 4C) and 75% for CLT (Figure 4D). The apparent Ki values calculated from these binding curves were 1.05±0.07 µM for TMX and 127±42.5 nM for CLT (Table 3). The residual binding could be explained by the inability of TMX and CLT to interact with all the nAChR subtypes recognized by IMI, or by an interaction of these ligands that were not strickly competitive with IMI.

### Toxicological effects of neonicotinoids on first-instar aphid larvae

**Figure 5:**
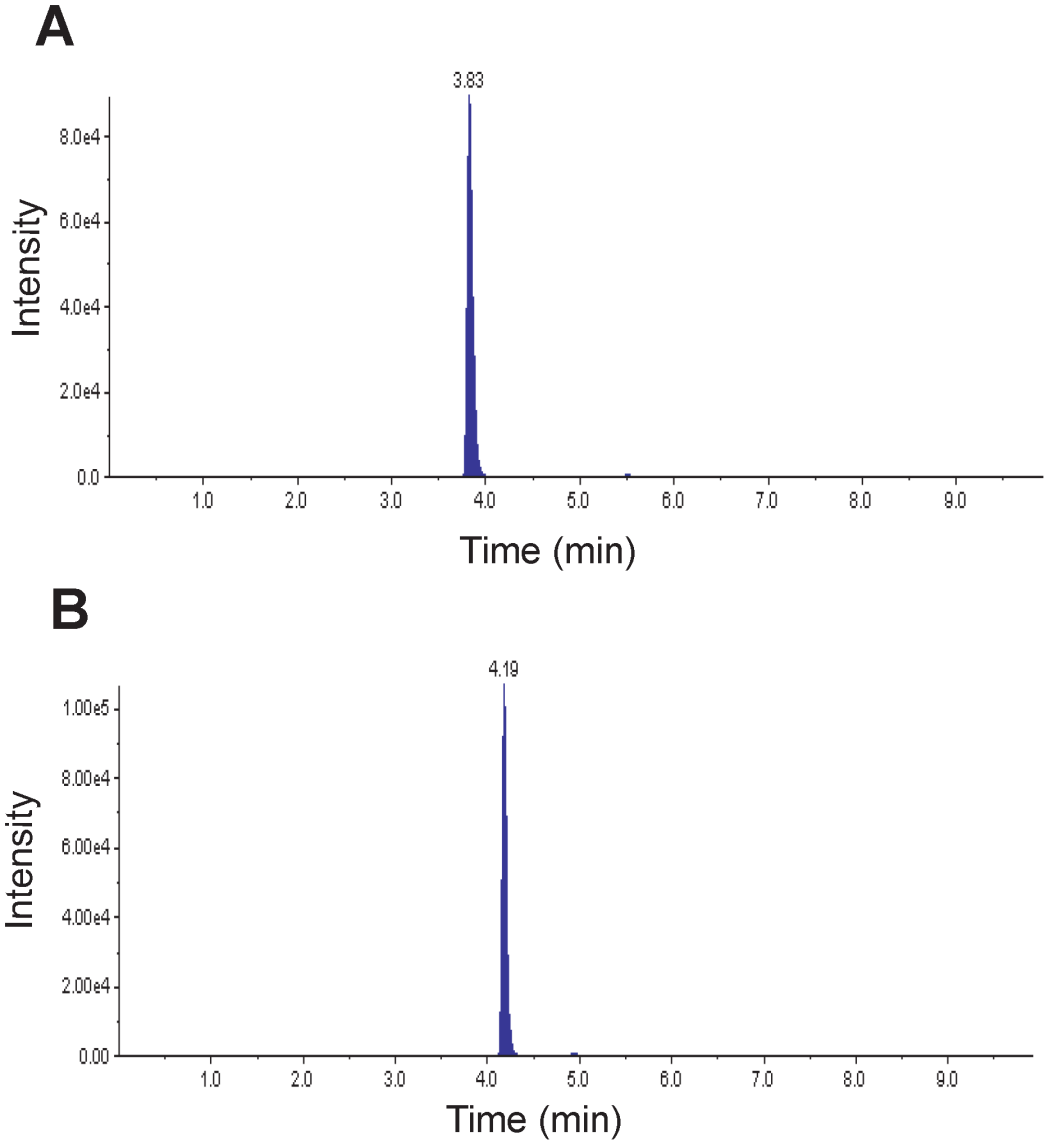
MRM chromatograms. Chromatograms of thiamethoxam (A) and its metabolite clothianidin (B) in 13,400 pea aphid larvae exposed to thiamethoxam at LC_50_ for 48 h. Intensity represents the peak area of the detected signal.

The toxicological effects of neonicotinoids upon *A. pisum* larvae have been previously studied [16]. Using the same method, we found that the three neonicotinoids TMX, CLT, and IMI have different toxicities against *A. pisum*. All results are presented in table 4 as means of 6 to 8 experiments. We found that TMX was the most toxic (LC_50_  =  0.259 µg/ml) and CLT was the least toxic (LC_50_  =  3.458 µg/ml) after 24 h of exposure. The toxicity of IMI was intermediate with an LC_50_ of 0.913 µg/ml. The LC_50_ values were significantly lower after 48 h of exposure. Interestingly IMI and TMX showed similar effect (LC_50_  =  0.038 and 0.034 µg/ml, respectively) whereas CLT remained the least toxic (LC_50_  =  0.118 µg/ml). We suggest that the potency of TMX could be associated to its double action: directly and after metabolization to CLT as previously proposed [25]. Indeed HPLC/MS analysis showed that TMX was metabolized to CLT (Figure 5). In TMX-treated aphids (using TMX at LC_50_  =  0.034 µg/ml) we obtained final concentrations of 1.34 µg/kg of TMX and 1.76 µg/kg of CLT, after 48 h of exposure.

**Table 4:** Neonicotinoid toxicity in the pea aphid on first-instar larvae for 24 h and 48 h of insecticide exposure using an artificial diet system.

	24 h		48 h		
	LC 50 (μg/ml)	CI 95%	LC 50 (μg/ml)	CI 95%	n
Imidacloprid	0.913 ^a^	0.266 – 3.133	0.038 ^d^	0.023 – 0.064	2381
Thiamethoxam	0.259 ^b^	0.039 – 1.718	0.034 ^d^	0.012 – 0.101	2613
Clothianidin	3.458 ^c^	0.834 – 14.34	0.118 ^e^	0.009 – 1.62	3016

n  =  number of insects tested; CI  =  confidence interval; LC_50_  =  Lethal concentration leading to 50% mortality. Results were corrected using Henderson-Tilton's formula. Values followed by different letters are significantly different (P<0.05; t-test with Welch's correction). Toxicity curves were determined with 8 concentrations and 6 to 8 replicates were made for each concentration.

### Is the expression of aphid nAChR subunits influenced by developmental stage or by exposure to neonicotinoids?

**Figure 6:**
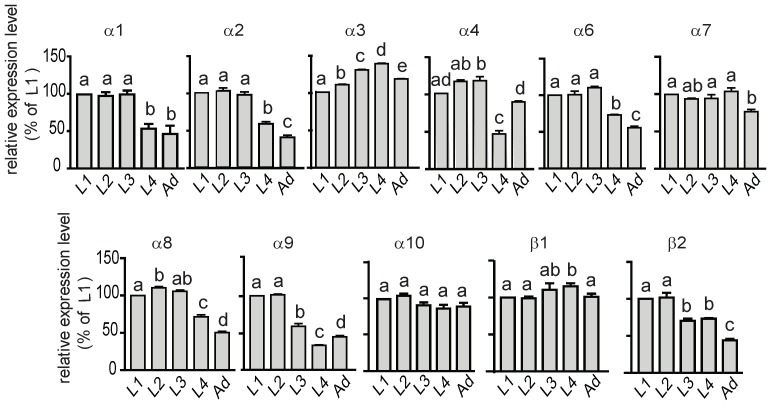
Expression level of nAChR mRNA subunits according to developmental stages of the pea aphid. Quantitative experiments were performed on whole individuals in triplicate. Results are mean of three independent experiments. Relative expression ratio were calculated relative to first-instar nymphs and normalized with external reference gene *luciferase*. Statistical analysis (One-Way ANOVA) was carried out using GraphPad Prism 5 software. For each subunit, expression ratio statistically different according to larval stage are designated by different letters.

Recently, using the full genome of *A. pisum*, Dale et al. highlighted the presence of 11 genes encoding putative nAChR subunits [15]. We confirmed the expression of these 11 genes in the pea aphid and studied the expression profile of these subunits according to the developmental stage and neonicotinoid exposure. First, qPCR experiments on the different developmental stages demonstrated that the expression of Apisumα1, Apisumα2, Apisumα6, Apisumα8 and Apisumβ2 was stable at the beginning of aphid development and then was significantly reduced during adulthood (One Way Anova, p<0.05, n = 3 experiments in triplicate, figure 6). On the contrary Apisumα3 expression increased with developmental stages although Apisumα7, Apisumα10 and Apisumβ1 transcript levels remained stable. The expression level of Apisumα4 and Apisumα9 subunits showed greater variability, with a lower expression level at the fourth larval stage. Thus, *A. pisum* subunits expression was regulated during developmental stages, suggesting that different nAChR subtypes could be expressed.

**Figure 7:**
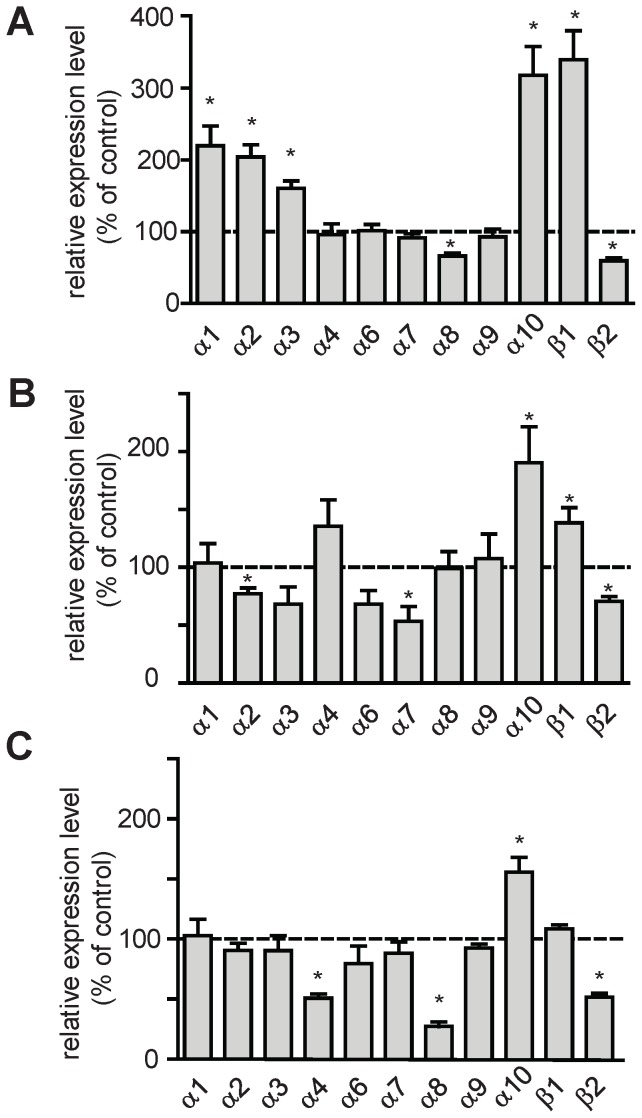
Expression levels of nAChR mRNA subunits after neonicotinoid exposure. Experiments were assessed on whole survivingl larvae exposed to neonicotinoids at LC_50_ for 48h. Aphids were intoxicated with imidacloprid (A) thiamethoxam (B) or clothianidin (C). Each qPCR experiment was performed in triplicate and results are represented as the mean of four to seven independent experiments after normalization with actin and ribosomal *rpl7* gene. Error bars represent the SEM. Results are expressed in % of the expression level in control conditions (no insecticide, corresponding to 100%). Statistical analysis (t-test, α = 0.05) was carried out using Graphpad Prism 5 software.

Second, qPCR experiments were performed on surviving aphid larvae exposed to IMI, TMX and CLT after 48 h exposure. For this purpose, aphid larvae were intoxicated at the LC_50_ determined in insecticidal assays. We found that IMI induced a strong variation of nAChR subunits expression compared to control condition, with the exception of Apisumα4, Apisumα6, Apisumα7 and Apisumα9 (Figure 7, n = 4 to 7 experiments in triplicate). We also observed a significant increase of Apisumα10 (+218±40%), Apisumβ1 (+240±40%), Apisumα1 (+120±27%), Apisumα2 (+104±17%) and Apisumα3 (+61±10%), respectively. On the contrary, a decrease was found with Apisumα8 (−34±4%) and Apisumβ2 (−40±4%), respectively (Figure 7A). Aphid larvae treated with TMX showed a significant decrease of Apisumα2 (−23±5%), Apisumα7 (−46±13%), Apisumβ2 (−29±4%) and an increase for Apisumα10 (+90±31%) and Apisumβ1 (+39±13%; figure 7B). Exposure to CLT led to a significant diminution of Apisumα4 (−49±4%), Apisumα8 (−73±4%) and Apisumβ2 (−48±3%) whereas we found a significant increase of Apisumα10 (+56±12%; figure 7C). These data confirmed that the expression of aphid nAChR subunits was differentially modified after exposure to various neonicotinoids.

## Discussion

### The pea aphid presents several pharmacological binding sites with different affinity for neonicotinoids

In the present studies, saturation binding experiments demonstrated that [^3^H]-IMI and [^125^I]-α-Bgt labeled high- and low-affinity nAChR binding sites in *A. pisum*. Binding properties of α-Bgt were not well documented in insects but two binding sites have also been found in the aphid *M. persicae* [13]. Interestingly, two specific [^3^H]-IMI binding sites were likewise reported in the aphids *M. persicae* and *A. craccivora* [11], [12]. In the pea aphid, the large difference between Bmax values for high and low affinity binding sites for both [^3^H]-IMI and [^125^I]-α-Bgt was in accordance with the presence of these sites on distinct nAChR subtypes and not at various subunit interfaces on the same receptor. In addition, competitive data showed that [^3^H]-IMI low affinity binding site was insensitive to α-Bgt. We proposed that IMI could bind to α-Bgt-insensitive nAChR subtypes, which was consistent with data obtained in *M. persicae* and *A. craccivora* [12]. Moreover, in the pea aphid, it seemed that the majority of binding sites was sensitive to α-Bgt, as previously demonstrated in *D. melanogaster* and *M. persicae* [11], [12], [13]. Competitive experiments also revealed that CLT bound to both [^125^I]-α-Bgt and [^3^H]-IMI binding sites. CLT was known to interact well with IMI-binding sites in the aphids *M. persicae* and *A. craccivora* [3], [26]. Interestingly, only one study referred to competitive experiments between CLT and labeled α-Bgt. Zhang et al. demonstrated a weak inhibitor potency of CLT to [^3^H]-α-Bgt binding sites in *D. melanogaster* [27]. The apparent discrepancy between these results and ours could be attributed to species specificity. Furthermore, TMX, which was metabolized to CLT, showed a weak binding capacity for [^3^H]-IMI binding sites and a better binding potency for [^125^I]-α-Bgt binding sites in *A. pisum*. These results are consistent with previous studies describing a lack of TMX competition with [^3^H]-IMI in other aphid species such as *M. persicae* and *A. craccivora* [3], [12]. Unfortunately, there was no data on TMX competitive binding to α-Bgt sites, despite that [^3^H]-TMX could bind directly in *M. persicae* and *A. craccivora* [28]. We propose that TMX binds to α-Bgt-binding sites in the pea aphid and that this mechanism could be present in other aphid species.

### The neonicotinoids IMI, TMX and CLT have different toxicological effects on *A. pisum*

Acute toxicological assays demonstrated that TMX and IMI were more toxic than CLT. Similar data have been found with *A. gossypii* in which IMI was more toxic than both TMX and CLT [10]. Interestingly, in *M. persicae* CLT was found to be a more potent insecticide than IMI [8]. This discrepancy could be linked to variation in the intoxication method and/or susceptibility of aphid species. In other studies, the neonicotinoid susceptibility was evaluated using topical application and a dipping method for *M. persicae* and *A. gossypii*, respectively [8], [10]. By contrast, we used an artificial diet protocol previously described by Sadeghi et al. [16]. The LC_50_ at 48 h for IMI corresponded to the LC_50_ at 72 h in Sadeghi's study which indicated that the pea aphid strains could be differentially sensitive to neonicotinoids. Moreover, because part of TMX was metabolized to CLT, we propose that the unusually high toxicity of TMX in *A.pisum* compared to other aphid species, was associated to its metabolite CLT [8], [10]. This hypothesis has been previously demonstrated using the moth *Spodoptera frugiperda* and the cockroach *Periplaneta americana*. Indeed, in *S. frugiperda* and *P. americana*, TMX was metabolized 24 h after treatment [25], [29]. Nevertheless, in the present study, the proportion of metabolized TMX was different than previous studies [25], [29]. Thus, the high insecticidal effect of TMX on the pea aphid could be due to its double action, by acting on nAChRs sensitive to α-Bgt and IMI.

Dale et al identified 11 putative genes encoding nAChR subunits in the pea aphid genome among which three were divergent (Apisumβ2, Apisumα9 and Apisumα10) and did not belong to conserved subunit groups between insects species [15]. Using qPCR experiments on surviving larvae, we demonstrated that IMI, TMX and CLT significantly influenced nAChR subunit expression. For all neonicotinoids tested, we found that Apisum α10 was highly expressed after treatment. This subunit is an uncommon nAChR subunit lacking one cysteine in the Cys-loop and could be involved in distinct functional properties [15]. Previous studies performed with electric ray *Torpedo* demonstrated that α subunit lacking one cysteine in the Cys-loop could co-assemble to form functional receptors that are expressed at the membrane [30]. In *Torpedo* the lack of one cysteine also led to the loss of α-Bgt binding sites [30]. As neonicotinoids bind to α-Bgt binding sites in the pea aphid, we propose that increased expression of Apisumα10 subunit could likewise lead to increased expression of nAChR subtypes that are less sensitive to neonicotinoids. After neonicotinoid exposure, we also observed that Apisumβ1 was over-expressed after TMX and IMI exposure and Apisumβ2 under-expressed after treatment with the three insecticides. Thus, in the pea aphid we proposed that both Apisumβ1 and Apisumβ2 could be differently involved in the regulation of neonicotinoid sensitivity. Indeed, studies performed on the brown planthopper *Nilaparvata lugens* and the aphid *M. persicae* showed that β1 was part of the IMI binding sites. Mutation of arginine to threonine at position 81 in this subunit induced an increase of resistance against neonicotinoids [31], [32], [33]. Moreover, recent studies demonstrated that nAChR subunit expression level was associated to neonicotinoid sensitivity [34], [35]. For example, a decrease in Accβ1 and Accβ2 subunit expression in the Asiatic honey bee *Apis cerana* was described after IMI exposure [35]. Our results also demonstrated that neonicotinoid toxicity was associated with specific nAChR subunit regulation. For example, Apisumα10 and Apisumβ2 were always up- or down-regulated, following treatment with any of the three insecticides. On the contrary, we found that Apisumα7 and Apisumα4 were decreased only after exposure to TMX and CLT and the expression of Apisumα1 and Apisumα3 was increased after IMI exposure. These results suggest that some subunits could be involved in specific insecticide action. Thus, we suggest that high and low affinity binding sites could involved several nAChR subtypes.

## Conclusions

Previous studies conclude that nAChR subunits influence the pharmacological properties of nicotinic receptors and thus could modify the neonicotinoid sensitivity [36]. Our results demonstrated that pea aphid nAChR subunits were differentially expressed, first between developmental stages, as previously demonstrated in *Drosophila* and *Apis cerana cerana* [35], [37], [38], and also according to the neonicotinoid exposure. Neonicotinoid sensitivity could then be dependent on either physiological status and/or environmental conditions in the pea aphid. Moreover, using toxicological and binding studies, we highlighted differences in neonicotinoid sensitivity in the pea aphid as compared to other aphid species and strains [8], [10], [16]. Thus, the insecticide strategies against aphid pests should be optimized for each particular species. In the pea aphid, the role of divergent subunits Apisumα9, Apisumα10 and Apisumβ2 could be of particular interest to further understand the neonicotinoid mode of action.
